# Development of an IgY Antibody-Based Immunoassay for the Screening of the CYP2E1 Inhibitor/Enhancer from Herbal Medicines

**DOI:** 10.3389/fphar.2016.00502

**Published:** 2016-12-22

**Authors:** Zhihui Jiang, Xuemei Jiang, Cui Li, Huiting Xue, Xiaoying Zhang

**Affiliations:** ^1^College of Veterinary Medicine, Northwest A&F UniversityYangling, China; ^2^Chinese-German Joint Laboratory for Natural Product Research, Qinling-Bashan Mountains Bioresources Comprehensive Development C.I.C., College of Biological Science and Engineering, Shaanxi University of TechnologyHanzhong, China; ^3^College of Veterinary Medicine, Xinjiang Agricultural UniversityUrumqi, China

**Keywords:** cytochrome P4502E1 (CYP2E1), chicken egg yolk antibody (IgY), falconoid, alcohol, APAP

## Abstract

Cytochrome P450 (CYP) 2E1 is an important enzyme involved in the metabolism of many endogenous and exogenous compounds. It is essential to evaluate the expression of CYP2E1 in the studies of drug–drug interactions and the screening of drugs, natural products, and foodstuffs. The present work is a feasibility study on the development of immunoassays using a specific and sensitive chicken-sourced anti-CYP2E1 IgY antibody. Cloning, expression, and purification of a recombinant CYP2E1 (mice origin) protein were carried out. Anti-CYP2E1 IgY antibodies were generated by immunizing white Leghorn chickens with purified recombinant CYP2E1 protein and were purified by immune affinity chromatography. The IgY titer attained a peak level (≥1:128,000) after the fifth booster injection. For evaluation of the expression of CYP2E1 in different herbal treatment samples, the mice were treated by oral gavage for 3 days with alcohol (50% 15 mL/kg), acetaminophen (APAP, 300 mg/kg), *Cornus officinalis* extract (100 mg/kg), Alhagi-honey extract (100 mg/kg), *Apocynum venetum* extract (100 mg/kg), hyperoside (50 mg/kg), isoquercetin (50 mg/kg), 4-hydroxyphenylacetic acid (50 mg/kg), 3-hydroxyphenylacetic acid (50 mg/kg), and 3,4-hydroxyphenylacetic acid (50 mg/kg). The expression of CYP2E1 was determined by Western blot analysis, immunohistochemistry, ELISA, and immunomagnetic beads (IMBs) using anti-CYP2E1 IgY in liver tissue. The results showed that *C. officinalis* extract, Alhagi-honey extract, *A. venetum* extract, hyperoside, isoquercetin, and their xenobiotics 4-hydroxyphenylacetic acid, 3-hydroxyphenylacetic acid, and 3,4-hydroxyphenylacetic acid significantly decreased CYP2E1 levels. Alcohol and APAP treatments significantly increased CYP2E1 levels as analyzed with Western blot analysis, immunohistochemistry, and ELISA. The IMB method is suitable for large-scale screening, and it is a rapid screening (20 min) that uses a portable magnet and has no professional requirements for the operator, which makes it useful for on-the-spot analysis. Considering these results, the anti-CYP2E1 IgY could be applied as a novel research tool in screening for the CYP2E1 inhibitor/enhancer.

## Introduction

Cytochrome P450 (CYP) 2E1 is one of the key enzymes in the metabolism of numerous low molecular weight (<100 Da) endogenous compounds and many xenobiotics. This enzyme is highly induced by food or beverage constituents (alcohol), drugs (acetaminophen) (Hartman et al., 2015), and pollutants (styrene). CYP2E1 may eliminate potentially toxic compounds and, paradoxically, bioactive compounds in toxins or carcinogens. For example, CYP2E1 is induced during alcohol or acetaminophen (APAP) overdose and results in the formation of ROS and the increased generation of hydroxyl radicals (Jin et al., 2013; Hartman et al., 2015). Increased oxidative stress from induction of CYP2E1 can damage cellular and mitochondrial components, including mitochondrial DNA and cytochrome *c* oxidase (Demeilliers et al., 2002). An altered expression of CYP2E1 can influence the disposition of drugs in the body, causing altered plasma drug concentrations and leading to adverse drug reactions or treatment failures. The evaluation of CYP2E1 expression by different assays is the major focus of this article.

**FIGURE 1:**
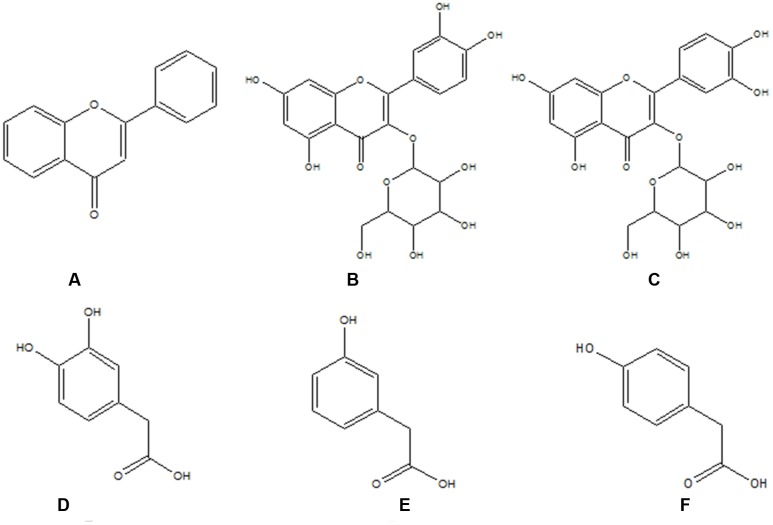
**The structures of flavonoids and their metabolites. (A)** Flavonoid skeleton; **(B)** hyperoside; **(C)** isoquercitrin; **(D)** 3,4-dihydroxy-phenylacetic acid; **(E)** 4-dihydroxy-phenylacetic acid; and **(F)** 3-dihydroxy-phenylacetic.

Flavonoids, a wide group of natural products, have gained considerable attention because of their low toxicity and powerful biological activities against many ailments, such as liver disease, cancer, reduced immune function, cardiovascular disease, and hypoglycemia. Flavonoids are widely distributed in plants and enter the human body through tea, vegetables, fruits, health care products, herbal medicines, and other plant-derived products. *Apocynum venetum* L., Alhagi-honey, and *Fructus Corni* are popular herbs in traditional Chinese medicine and are rich in flavonoids. Hyperoside and isoquercetin (**Figure 1**) are major flavonoids significant for their hepatoprotective effects, but they are poorly absorbed in the gastrointestinal tract (Chen et al., 2005). After flavonoid administration, colonic bacteria convert flavonoids into several simple phenolic acids – such as 4-hydroxyphenylacetic acid, 3-hydroxyphenylacetic acid, and 3,4-dihydroxybenzoic acid (**Figure 1**) (Yang et al., 2013) – that can be absorbed into the circulation and exert biological effects in the body. CYP2E1 are not only the metabolic enzymes directly involved in hepatic oxidative damage, but they also are important targets of the oxidation mechanism of liver injury. Flavonoids have strong antioxidant and hepatoprotective properties. Therefore, we speculated that flavonoids might be active compounds that can reduce the impact of liver-damaging substances by inhibiting the expression of CYP2E1, which might be one of the hepatoprotective mechanisms of flavonoids. Also, an investigation of the effects of flavonoids on CYP2E1 expression is necessary since some daily foods (purple potato, purple sweet potato, broccoli, bitter gourd, garlic, and tomato), beverages (coffee and cacao polyphenol), and drugs (*Schisandra chinensis*, danshen, *Gelsemium elegans*, isoniazid, diallyl sulfide, sulforaphane, and tamoxifen) are metabolized by CYP2E1 (Vang et al., 1991; Gu et al., 1992; Wargovich, 2006; Wang et al., 2010, 2015; Konstandi et al., 2013; Su et al., 2013; Ahmad et al., 2014; Lu et al., 2014; Zhou et al., 2014, 2015; Shayakhmetova et al., 2015; Suzuki et al., 2015; Jiang et al., 2016a,b).

The way to evaluate the influence of CYP2E1 on drug interactions is to examine the activity and protein expression of CYP2E1. The most common assay to test the activity of CYP2E1 is a PK-based method, such as HPLC (Martinez-Gil et al., 2015) and HPLC-MS/MS (Liang and Zheng, 2014). While these assays are effective in determining the activity of CYP2E1, they require substrates and costly instruments. Another method to test the expression of CYP2E1 is Western blotting. This assay, however, is a time-consuming (more than 6.5 h), complicated operation with many steps, expensive instruments, and no high-throughput screening. This article focuses on the development of a fast, sensitive, convenient, and immuno-based method to determine the expression of CYP2E1.

IgY is the major serum antibody in avians, amphibians, and reptiles. The significant advantage of using IgY antibodies is that egg collection is non-invasive compared to the stressful bleeding of animals to obtain serum. Also, the absence of immunological cross-reactivity between chicken IgY and mammalian IgG reinforces the advantages of using IgY over IgG as the antibody of choice in some types of immunological reactions (Larsson et al., 1993). For instance, in immunochemical analysis, cross-reactions are commonly observed between tissue IgG and epitopes shared by the primary antibody and recognized by the secondary mammalian antibody. This cross-reactivity is not observed when IgY is used as the secondary antibody (Dias da Silva and Tambourgi, 2010). Therefore, the aim of the present study is to develop an immunoassay to determine CYP2E1 expression in mammals. An anti-CYP2E1 antibody can be applied as an efficient research tool alternative to mammalian sourced antibodies. This study also focuses on investigating flavonoids and their secondary metabolites that may affect CYP2E1 expression and cause medicine/food-medicine interactions.

## Materials and Methods

### Cloning and Expression of CYP2E1

Total mRNA was extracted from a mouse liver using an RNAsimple Total RNA Kit (Tiangen Biotech, Beijing, China). The CYP2E1 gene was amplified using primers P-Forward (CGGGATCCGTTCTTGGCATCACCGTTGC) and P-Reverse (CCCAAGCTTCTATTGTAACAGGGCTGAGGTCG). The target gene was subcloned into a pET-32 vector according to NCBI sequence information (NM_021282.2), and the resulting plasmids were transformed into *E. coli* BL21. Individual colonies were selected and inoculated into 2YT broth that contained ampicillin, and were subsequently incubated overnight at 37°C. After OD 600 reached 0.6, IPTG was added, and the cultures were incubated for 10 h at 20°C to induce expression of CYP2E1. The pellets and supernatants were used to analyze the protein solubility by SDS-PAGE. The inclusion bodies were predicated by 50 mM Tris-HCl (pH 8.0) containing 3% SDS and 1 mM EDTA. The supernatant was collected by centrifugation at 12,000 rpm and added to 50 mM Tris-HCl (pH 8.0) containing 0.15 M NaCl, 1 mM EDTA, 5% glycerol, and 200 mM urea for 24 h. The protein was dialyzed against PBS for 2 days at 4°C. The purified protein was assessed by SDS-PAGE.

### Preparation of Anti-CYP2E1 IgY

#### Chicken Immunization

Seven-week-old white Leghorn chickens were first immunized with 250 μg of CYP2E1 protein with Freund’s complete adjuvant (FCA; Sigma-Aldrich Co., St. Louis, MO, USA). Three boosters with Freund’s incomplete adjuvant were injected at 2-week intervals, and the last booster with Freund’s incomplete adjuvant was injected at a 30-day interval. After the first booster immunization, eggs were collected daily, marked, and stored at 4°C until further processing. This study was carried out in accordance with the recommendations of Li et al. (2016), Ethics Committee of Northwest A&F University, the Guide for the Care and Use of Laboratory Animals.

Total IgY was extracted using PEG 6000, as described in He et al. (2015). In brief, each egg yolk was separated and diluted 1:2 with PBS (pH 7.4). To remove the lipids and lipoprotein, 3.5% PEG 6000 was added to the yolk. After 13,000 *g* centrifugation at 4°C for 20 min, the suspensions were filtered and 8.5% PEG 6000 was added for 30 min. The mixtures were centrifuged again at 13,000 *g* and 4°C for 20 min. The precipitate was dissolved in 10 mL PBS with 12% PEG 6000. The mixtures were centrifuged again in the same conditions as above. Finally, the precipitate (IgY extract) was dissolved in 1.2 mL PBS. The extract was contained in the dialysis capsule, dialyzed with PBS (1,000 mL), and gently stirred using a magnetic stirrer for 3 days at 4°C. The PBS was changed every 8 h.

Immunoaffinity chromatography was used to purify the anti-CYP2E1 antibodies. Purified recombinant CYP2E1 protein was subjected to SDS-PAGE under reducing conditions on 12% (80 V) and 5% gels (120 V). Separated proteins were then transferred to PVDF membranes (120 mA, 40 min). After using 5% milk as a blocking agent for 1 h, the PVDF membranes were incubated with the isolated anti-CYP2E1 IgY antibodies (1:1,000) on the orbital shaker for 2 h. Next the membranes were incubated with 1 mL Tris-HCl (pH 2.0) for 20 min, and then the same volume of Tris-HCl (pH 8.0) was added for neutralization. The purity of the anti-CYP2E1 IgY was then tested by SDS-PAGE.

### Monitoring of the IgY Titer

Indirect ELISA was used to determine the titer of the specific antibody as described (He et al., 2015). The optimal concentration of the agent was selected by the checkerboard method. Western blotting was employed to determine the purity and specificity of anti-CYP2E1 IgY; the protein was separated using 12% SDS-PAGE and transferred onto a PVDF membrane. The unreacted sites were blocked with 5% nonfat milk for 2 h at 37°C, then incubated with anti-CYP2E1-IgY (1:1,000) for 2 h and with an HRP-conjugated goat anti-chicken antibody (1:10,000; Sigma, MO, USA) for 1 h. The signal was visualized using ECL reagents from the ChemiDoc Imaging System (Tanon 3500, Shanghai, China).

### Flavonoid Extraction

Each 10 g of Alhagi-honey and *Corni fructus* (*Sieb. Et Zucc*) and 50 g of dried leaves of *A. venetum* L. were extracted twice with 300 mL of 70% ethanol in a refluxed condenser for 1 h each. The final volume was 100 mL of ethanol. The extracts were frozen, lyophilized to dryness (Alhagi-honey 2.7 g, *C. fructus* 3 g, and *A. venetum* L. 2.5 g), and stored at 4°C until the analyses.

### Total Flavonoid Assay

The total flavonoid content was measured based on a previously described method (HaiYing et al., 2009). Briefly, 0.5 mL of extract, 0.3 mL of 5% Na_2_NO_2_, and 0.3 mL of 10% AlCl_3_-methanol solution were mixed in a 10 mL test tube. After 6 min, 2 mL NaOH (1 M) solution was added, and then the mixture was eventually added to 10 mL of 50% v/v methanol. The absorption value was measured using a spectrophotometer at 510 nm. A standard curve for total flavonoids was prepared using rutin (0, 2.5, 5, 10, 20, 40, 80, and 120 mg/g), and the total flavonoid content was expressed as milligrams of rutin equivalents per gram of dried extract.

### Animal Treatments

A total of 4-week-old Kunming female and male mice were purchased from the animal center of the Fourth Military Medical University, Xi’an, China. The mice had access to drinking water and food and were housed at room temperature with an automatic 12 h light and 12 h dark cycle. All animal experiment protocols were reviewed and approved by the Ethics Committee of Northwest A&F University for the use of Laboratory Animals.

After a 1-week acclimation, the mice were randomly divided into 11 groups, seven in each group. All the treatment groups were treated by oral gavage for 3 days as per the experimental design. The 11 groups were: a normal group (normal saline 10 mL/kg), APAP group (APAP 300 mg/kg), alcohol group (50% 15 mL/kg), *Cornus officinalis* group (100 mg/kg extraction), Alhagi-honey group (100 mg/kg extraction), *A. venetum* group (100 mg/kg), hyperoside group (50 mg/kg), isoquercetin group (50 mg/kg), 4-hydroxyphenylacetic acid group (50 mg/kg), 3-hydroxyphenylacetic acid group, (50 mg/kg), and 3,4-hydroxyphenylacetic acid group (50 mg/kg extraction).

The mice were sacrificed with diethyl ether 24 h after the last administration. Their livers were immediately removed. One part of the liver was fixed with 4% paraformaldehyde; the other part was washed in ice-cold saline and frozen at –20°C for further study.

### Western Blot Analysis of CYP2E1 Protein Expression

Liver microsomal protein preparation was carried out per the previous study by Jiang et al. (2016a). After determining the protein concentration using the Bradford protein assay (Tiangen Biotech, Beijing, China), protein samples (80 μg) were run on a 12% and 5% SDS-PAGE gel and transferred onto PVDF membranes. After blocking, the membranes were incubated with anti-CYP2E1 IgY antibodies (1:1,000) and mouse polyclonal anti-GAPDH antibodies (1:1,000; Wuhan Boster Biological Technology, Wuhan, China) for 2 h. The membrane was washed three times with TBST and incubated with an HRP-conjugated rabbit anti-chicken IgY antibody (1:10,000, Sigma, USA) or a goat anti-mice IgG antibody (1:10,000; Wuhan Boster Biological Technology, Wuhan, China) for 2 h. The signal was visualized using the ChemiDoc Imaging System (Tanon 3500, Shanghai, China).

### Indirect ELISA Determination of CYP2E1 Protein Expression

Indirect ELISA was used to detect the expression of CYP2E1 in each treated sample. The total sample protein was coated onto the 96-well plates overnight at 4°C, and 5% non-fat milk was used for blocking for 2 h at 37°C. After washing three times with PBS, the purified anti-CYP2E1 IgY antibody (1:1,000) and the commercial anti-CYP2E1 IgG antibody were incubated for 2 h at 37°C. After washing three times with PBST and one time with PBS, the secondary goat anti-chicken IgG conjugated to HRP (1:10,000, Sigma, USA) was added for 1.5 h at 37°C. After washing, TMB was added and incubated for 5 min in the dark at room temperature, and the absorbance was read at 450 nm.

### Immunohistochemistry Investigation on CYP2E1 Location and Expression

The fixed mice liver tissue was embedded in paraffin and sectioned at 5 μm thickness. The sections were incubated with anti-CYP2E1 IgY antibody (1:800) for 1 h, then washed three times using PBS and incubated with rabbit anti-chicken secondary antibody (1:10,000, Sigma, USA) for 1 h. After the sections were washed with PBS three times, the images were captured using a digital camera.

### Immunomagnetic Aggregation Sensor Screening for CYP2E1 Protein Expression

The immunomagnetic beads (IMBs) were employed as described in Chen et al. (2016). Briefly, the amine IMBs (Emerther Company, Shanghai, China) were activated by 5% glutaraldehyde and a coupling buffer for 3 h. Subsequently, 0.1 mg anti-CYP2E1 IgY antibody was added to the activated IMB solution. The mixture was gently stirred, to allow the reaction to proceed, for 24 h at room temperature and then blocked with 1% BSA for 0.5 h. The IMBs were suspended with washing buffer and stored at 4°C for further use.

To detect CYP2E1 expression, researchers transferred 300 μL of the IMB solution and 1,200 μL of the microsomal protein to centrifuge tubes. Each mixture was incubated for 20 min. Then, all the tubes were put on a magnetic scaffold, the color changes of the IMBs were observed after 1 min, and the images were captured by a digital camera. The colors of the IMBs were processed into grayscale values by ImageJ software (National Institutes of Health, MD, USA).

To detect the specificity of immunomagnetic aggregation (IMA) sensor, the protein in the magnetic beads were eluted with 1 mL Tris-HCl (pH 4.5) for 20 min, and then the same volume of Tris-HCl (pH 8.0) was added for neutralization. The elution was run on 12 and 5% SDS-PAGE gels and transferred onto PVDF membranes. After blocking, the membranes were incubated with anti-CYP2E1, CYP1A2, CYP2C9, CYP2D6, CYP2C19, and CYP3A4 IgG antibodies (1:1,000; Bioss, Beijing, China) for 2 h. The membrane was washed three times with TBST and incubated with an HRP-conjugated rabbit anti-IgG antibody (1:2,000, Senjian, Tianjing, China) for 2 h. The signal was visualized using the ChemiDoc Imaging System.

### Data Analysis and Statistical Methods

The data were analyzed using SPSS 19 software (SPSS Inc., Chicago, IL, USA). A one-way ANOVA was employed for comparison among the groups, and Tukey’s comparison tests of significant differences among groups were utilized. The results were expressed as mean ± standard deviation (*SD*) using Graph Pad Prism Software v.7 (GraphPad Software, Inc., San Diego, CA, USA).

## Results

### CYP2E1 Gene Amplification and Anti-CYP2E1 IgY Antibody Purification

**FIGURE 2:**
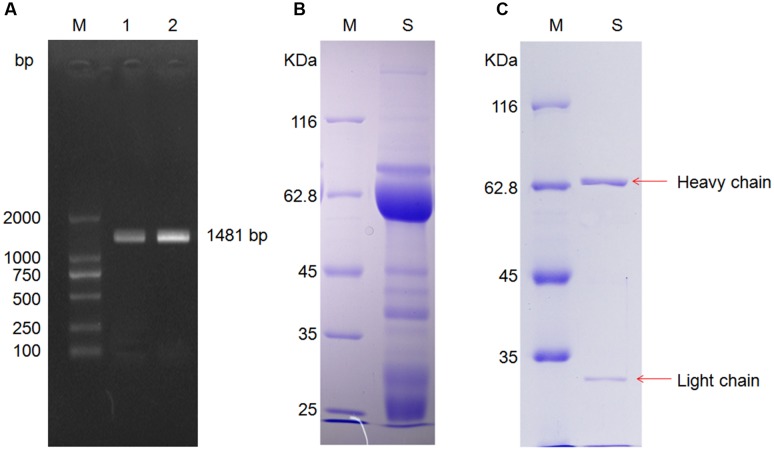
**Cytochrome P4502E1 (CYP2E1) gene amplification and anti-CYP2E1 IgY antibody purification. (A)** Lane M: marker, lane 1 and 2 are amplification of CYP2E1; **(B)** lane M: marker, lane S: unpurified anti-CYP2E1 IgY antibody; and **(C)** lane M: marker, lane S: purified anti-CYP2E1 IgY antibody.

The total of mRNA in mice liver was extracted, the cDNA fragment of the CYP2E1 gene was PCR-amplified by PCR, and the 1,481 bp product (**Figure 2A**) was cloned into the recombinant expression vector pHT-32a-CYP2E1, which was then transformed into *E. coli* BL21 to express the CYP2E1 protein.

After PEG 6000 extraction, the IgY light chain, heavy chain, and some lower molecular weight bands were observed in SDS-PAGE under reducing conditions (**Figure 2B**). The lower molecular weight bands were removed by immunoaffinity chromatography (**Figure 2C**).

### Titer and Binding Activity of Anti-CYP2E1 IgY

**FIGURE 3:**
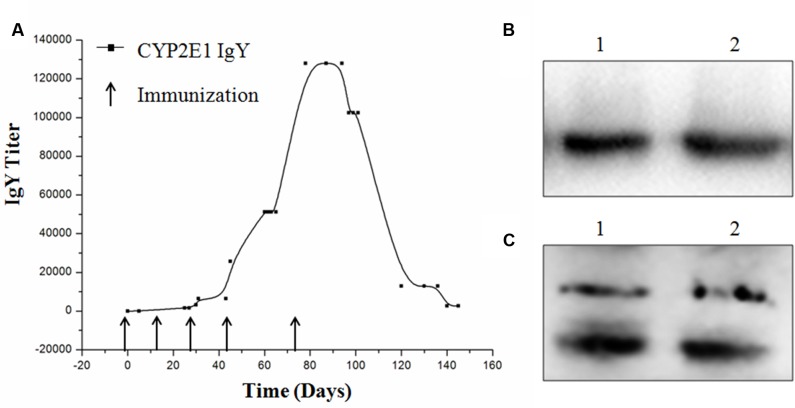
**Titer and binding activity of anti-CYP2E1 IgY. (A)** Development of IgY titer against recombinant CYP2E1 protein; **(B)** lane 1 and 2, CYP2E1 protein recognized by IgY; and **(C)** lane 1 and lane 2, CYP 2E1 protein recognized by commercial IgG polyclonal antibody.

The titers of specific IgY were determined by indirect ELISA. The IgY titer (1:25,600) was increased after the fourth booster injection, reached a peak (1:128,000) after the fifth booster injection, and was found to be stable at the highest titer for more than 1 month. The titers gradually decreased to 1:25,600 and remained at this level until the end of the experiment (**Figure 3A**). The binding activity of IgY to CYP2E1 was further determined by Western blot analysis. The isolated IgY was bound to the CYP2E1 protein at a dilution of 1:1,000 (**Figure 3B**). The commercial IgG antibody was bound to three proteins at a dilution of 1:1,000 (**Figure 3C**).

### Total Flavones of Herbal Extracts

The values of standard rutin showed an excellent linear relation, *y* = 3.907*x*+0.0080 (*R*^2^ = 0.9991). The total flavone contents of *A. venetum* L. extract, Alhagi-honey extract, and *C. officinalis* extract were found to be 62.2 ± 0.8 mg/g, 41.3 ± 0.4 mg/g, and 52.1 ± 0.7 mg/g, respectively.

### Western Blot and Immunohistochemistry Determination of the Effect of Different Flavone Treatments on CYP2E1

**FIGURE 4:**
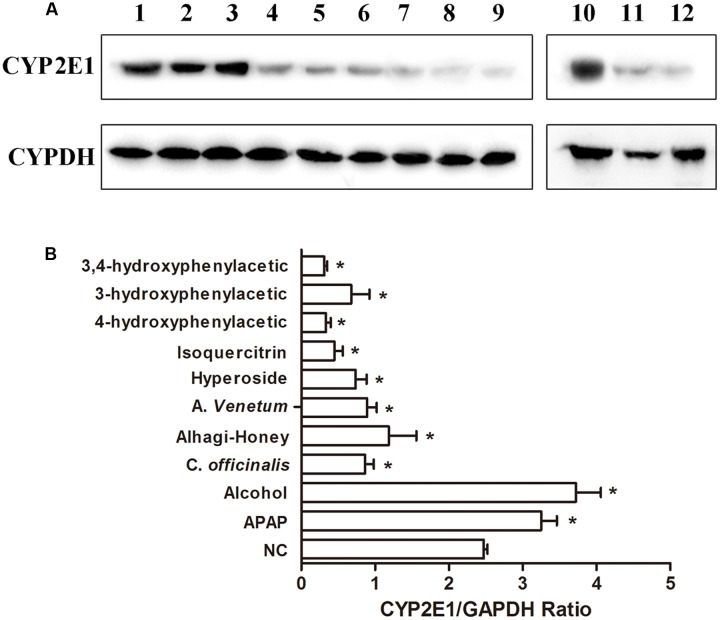
**Western blot analysis of CYP2E1 expression using CYP2E1 IgY. (A)** Representative blots of CYP2E1 and GAPDH expression. lane 1, control group; lane 2, APAP-treated group; lane 3, alcohol-treated group; lane 4, *Cornus officinalis*-treated group; lane 5, Alhagi-honey-treated group; lane 6, *Apocynum venetum*-treated group; lane 7, hyperoside-treated group; lane 8, isoquercitrin-treated group; lane 9, 4-hydroxyphenylacetic-treated group; lane 10, control group; lane 11, 3-hydroxyphenylacetic-treated group; lane 12, 3,4-hydroxyphenylacetic-treated group. **(B)** Relative quantification of CYP2E1 protein levels by Gel-Protein analyzer soft. Each value represents the mean ± *SD* of three independent experiments. ^∗^*P* < 0.05 compared with the control group.

**FIGURE 5:**
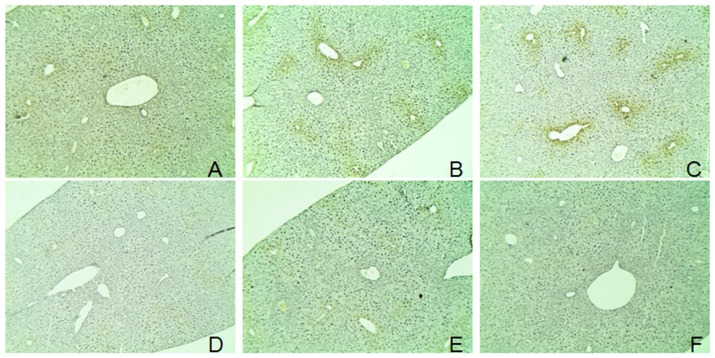
**Immunohistochemical detection of CYP2E1 expression.** The mice livers were used for immunohistochemical detection. **(A)** control group; **(B)** alcohol-treated group; **(C)** APAP-treated group; **(D)** hyperoside-treated group; **(E)** isoquercitrin-treated group; and **(F)**, 3,4-hydroxyphenylacetic-treated group.

The protein expressions of CYP2E1 were significantly increased in APAP- and alcohol-treated groups (**Figures 4A,B**), and the ratios of protein expression of CYP2E1 in alcohol and APAP-treated groups were 1.5- and 1.31-fold higher than that of the control group. The expression of CYP2E1 in *C. officinalis* extract, Alhagi-honey extract, and *A. venetum* L. extract was significantly lower than that of the controls. The isoquercetin, hyperoside, 4-hydroxyphenylacetic acid, 3-hydroxyphenylacetic acid, and 3,4-hydroxyphenylacetic acid treatments significantly reduced CYP2E1 expression, and the levels of CYP2E1 were the lowest in the 3,4-hydroxyphenylacetic acid and 4-hydroxyphenylacetic acid treatments among all the treatments. The location of CYP2E1 expression was determined by immunohistochemistry. As shown in **Figure 5**, CYP2E1 expression was located around the central vein in the mouse liver and was induced by alcohol and APAP treatments. Hyperoside, isoquercetin, and 3,4-hydroxyphenylacetic acid significantly decreased the CYP2E1 expression.

### ELISA Assessment on the Effect of Different Flavone Treatments on CYP2E1

**FIGURE 6:**
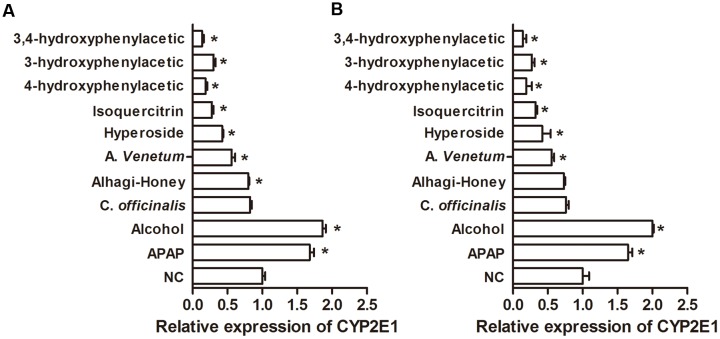
**Relative expression of CYP2E1 in different treatments. (A)** Using anti-CYP2E1 IgY antibody; **(B)** using anti-CYP2E1 IgG antibody. ^∗^*P* < 0.05 compared with the control group.

The hepatic expression of CYP2E1 in different treatments was detected by direct ELISA with an anti-CYP2E1 IgY or IgG antibody. Alcohol and APAP increased the levels of CYP2E1, as shown in **Figure 6**, and the Alhagi-honey extract, *A. venetum* extract, isoquercetin, hyperoside, 4-hydroxyphenylacetic acid, 3-hydroxyphenylacetic acid, and 3,4-hydroxyphenylacetic acid significantly decreased the expression of CYP2E1. The expression of CYP2E1 in the *Cornus* Alhagi-honey extract was significantly decreased with control group as detected by the IgY antibody, and Alhagi-honey extract treatment using an IgG antibody were not significantly different compared with control group.

### IMB Evaluation of the Effect of Different Flavone Treatments on CYP2E1

**FIGURE 7:**
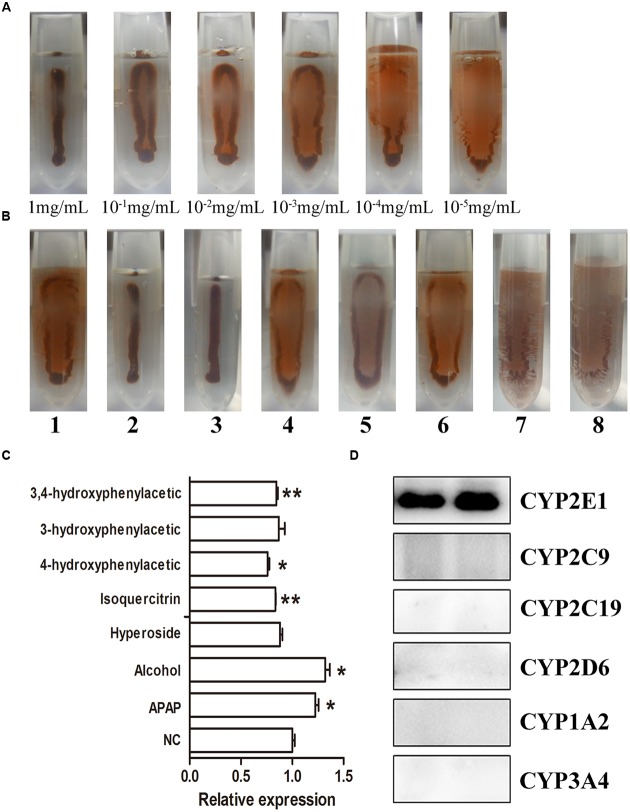
**IMA sensor detection of CYP2E1 expression. (A)** Different concentrations of CYP 2E; **(B)** the expression of CYP2E1 in different treatment samples. 1, control; 2, APAP; 3, alcohol; 4, isoquercitrin; 5, hyperoside; 6, 4-hydroxyphenylacetic; 7, 3-hydroxyphenylacetic; 8, 3,4-hydroxyphenylacetic; **(C)** relative densitometry intensity of IMA sensor signal as assessed by ImageJ; **(D)** cross-reactivity of anti-CYP2E1 with various anti-CYP450 antibodies. ^∗^*P* < 0.05 and ^∗∗^*P* < 0.01 compared with the control group.

Using IMA sensors, the color intensity changed from dark brown to light yellow when the concentration of the IMBs changed from 1 to 10^–5^ mg/mL (**Figure 7A**). As shown in **Figure 7B**, IMBs in samples 2 and 3 were heavily aggregated, samples 1, 4, and 6 were gently aggregated, and the other samples were well-dispersed, suggesting that the CYP2E1 protein expression was significantly increased (*p* < 0.05) in APAP- and alcohol-induced groups. The isoquercetin, hyperoside, 4-hydroxyphenylacetic acid, 3-hydroxyphenylacetic acid, and 3,4-hydroxyphenylacetic acid treatment significantly reduced CYP2E1 expression (**Figure 7C**), and the trend of expression was consistent with the Western blot and ELISA results. The cross-reactivity of the anti-CYP2E1 IgY antibody with the anti-CYP1A2, CYP2C9, CYP2D6, CYP2C19, and CYP3A4 antibodies was tested by Western blot. Results indicated that the anti-CYP2E1 IgY antibody showed no cross-reactivity with other proteins (CYP1A2, CYP2C9, CYP2D6, CYP2C19, and CYP3A4), indicating that the anti-CYP2E1 IgY antibody is highly specific for CYP2E1 (**Figure 7D**).

## Discussion

The CYP2E1 level is a key indicator in pharmacological and toxicological screening. The classical assay to assess the CYP2E1 protein and activity is by PK-based HPLC and HPLC-MS/MS analysis. While these assays are precise and sensitive methods for CYP2E1 detection, CYP2E1-specific substrates and sophisticated instrumentation are required for analysis. In this study, we have developed an alternative analysis immunochemical procedure that is fast and sensitive for CYP2E1 screening. The anti-CYP2E1 antibody was successfully prepared, and the levels of hepatic CYP2E1 expression in different flavonoid treatment groups, as well as alcohol and APAP treatments, were evaluated by different immunoassays.

The IMB assay is a novel biosensor assay that combines magnetic separation and a visual readout in one step for the determination of biomarkers (Chen et al., 2016). Quantification of the protein being detected is dependent on the degree of IMB aggregation, which is sample-concentration dependent. Compared to other immunoassays, the IMB assay enables readout by the naked eye (Chen et al., 2016). The aggregated IMBs will present a brown color with a narrow stripe, distinguishable from dispersed IMBs with a yellow coating. In this study, the color change of IMBs could allow the reader to distinguish concentrations of CYP2E1 ranging from 10^–5^ mg/mL to 1 mg/mL (**Figure 7A**). Results indicated that the anti-CYP2E1 IgY antibody showed no cross-reactivity with other proteins (CYP1A2, CYP2C9, CYP2D6, CYP2C19, and CYP3A4), indicating that the anti-CYP2E1 IgY antibody is highly specific for CYP2E1; however, the sensitivity of IMB is lower than that for ELISA and Western blotting. Importantly, our IMB tendency is consistent with those using Western blotting, and this semi-quantity is sufficient for preliminary analysis of herbs and compounds. Compared to Western blotting, the IMB method is suitable for large-scale screening, as it is a rapid process (20 min) that applies a portable magnet and does not require a professional for its operation. These features make IMB suitable for on-the-spot analysis. Western blot and ELISA are widely used methods for the immunological detection of proteins. Compared to IgG antibodies, using anti-CYP2E1 IgY antibodies creates a significant difference between each group by ELISA (**Figure 6**). For example, the levels of CYP2E1 in Alhagi-honey were significantly different from that of the control using the anti-CYP2E1 IgY, while no significant difference was observed using the anti-CYP2E1 IgG, suggesting the anti-CYP2E1 IgY antibody has a good sensitivity and specificity. However, these immunoassays are time-consuming and require complex manipulation and expensive instrumentation. The IMB assay is easily developed into a rapid assay, allowing a one-step process that can be completed within 20 min. IMB is a low-cost and portable diagnostic tool with magnetic beads and a camera, thus meeting the needs of point-of-care-testing and high-throughput screening. Furthermore, IMBs are stable after 24 h in a magnetic field without affecting the results of the IMA sensor (Chen et al., 2016).

Chickens are a good choice for the preparation of specific antibodies with high titers developed against highly conserved mammalian antigens due to the evolutionary distance between the source of the antigen (mammals) and the immune system (chickens) (Schade and Hlinak, 1995). Indeed, it has been widely recognized that the absence of immunological cross-reactivity between chicken IgY and mammalian IgG is determined by the evolutionary divergence (Kovacs-Nolan and Mine, 2004). In this study, the highest titer of 1:1,280,000 was observed after the fifth booster injection, and it remained elevated for more than 1 month (**Figure 3**). IgY is easily produced with large yields of approximately 100 mg IgY per egg yolk (Schade and Hlinak, 1995). A total of 84 eggs were collected with anti-CYP2E1 IgY titers of more than 1:25,600 CYP2E1 per egg yolk following four boosters. Western blotting revealed that the anti-CYP2E1 IgY antibody could specifically recognize the CYP2E1 protein. The anti-CYP2E1 IgY antibody showed a lower false positive rate than that of a commercial antibody (**Figures 3B,C**). These findings suggest that anti-CYP2E1 is a valuable research tool for exploring the expression of the CYP2E1 protein.

An overdose of alcohol or APAP generates the *first hit* on the liver, and the major metabolic enzyme CYP2E1 could generate the *second hit* of cellular injury (Robertson et al., 2001), particularly when antioxidant reserves are depleted. In this study, higher levels of CYP2E1 expression were observed after alcohol or APAP treatment by different immunoassays. In contrast, *C. officinalis* extract, Alhagi-honey extract, and *A. venetum* extract significantly decreased the expression of CYP2E1. These three herbal medicines can protect the liver against alcohol- or APAP-induced liver injuries in animal models (Shaker et al., 2010; Nam et al., 2014; Xie et al., 2015), suggesting that hepatocyte damage was effectively attenuated by *C. officinalis* extract, Alhagi-honey extract, and *A. venetum* extract. To further explore whether the major hepatoprotective effects of flavonoids and their metabolites can inhibit the expression of CYP2E1, hyperoside, isoquercetin, 4-hydroxyphenylacetic acid, 3-hydroxyphenylacetic acid, and 3,4-hydroxyphenylacetic acid were used to modify the expression of CYP2E1 as determined by Western blot, immunohistochemistry, ELISA, and IMB methods. Results showed that these five compounds all inhibited the expression of CYP2E1, suggesting they may protect the liver against alcohol- and APAP-induced injury. The clinical dosages of drugs metabolized by human CYP2E1 might need to be adjusted in patients because these herbal medications may increase the plasma concentrations of these drugs.

## Conclusion

In this study, a rapid IMB was developed to test the hepatic expression of CYP2E1 following different flavonoid treatments. This investigation formed the basis for a proof of concept for the use of various applications of anti-CYP2E1 IgY as a screening tool using different immunological methods. Also, the use of anti-CYP2E1 IgY with IMA sensors could be applied as a fast and useful research tool in the development and high-throughput screening of CYP2E1 inhibitor/enhancer compounds by the naked eye based on the color change of the IMBs. Furthermore, this assay may serve as a valuable tool to provide an application for the detection of clinical drug–drug interactions observed during early or late drug development.

## Ethics Statement

All animal experimental protocols were reviewed and approved by the Ethics Committee of Northwest A&F University for the use of Laboratory Animals. (1) Mice – a total of 4-week Kunming female and male mice were purchased from the animal center of the Fourth Military Medical University, Xi’an, China. Mice were allowed to freely access drinking water and food and were housed at room temperature with an automatic 12 h light and 12 h dark cycle. After 1-week acclimation, the mice were randomly divided into 10 groups, seven in each group. All the treatment groups were treated by oral gavage for 3 days as per following experimental design. Normal group (normal saline 10 mL/kg), APAP group (APAP 300 mg/kg), Alcohol group (50% 15 mL/kg), *C. officinalis* group (100 mg/kg extraction), Alhagi-honey group (100 mg/kg extraction), *A. venetum* group (100 mg/kg), hyperoside (50 mg/kg), isoquercitrin (50 mg/kg), 4-hydroxyphenylacetic acid (50 mg/kg), 3-hydroxyphenylacetic acid (50 mg/kg), and 3,4-hydroxyphenylacetic group (50 mg/kg extraction). The mice were sacrificed 24 h with diethyl ether after the last administration. Livers were instantly removed, one part of the liver was fixed with 4% paraformaldehyde, and the other part was washed in ice-cold saline and frozen at –20°C for further study. (2) Chicken immunization –7-week-old white Leghorn chickens were first immunized with 250 μg of CYP2E1 protein with Freund’s complete adjuvant (FCA; Sigma-Aldrich Co., St. Louis, MO, USA), four booster injections with Freund’s incomplete adjuvant were injected in 2-week intervals. The last booster with Freund’s incomplete adjuvant was injected at a 30-day interval. Eggs were collected daily after the first day booster-immunization, marked, and stored at 4°C until further processing. We state that we do not use minors, persons. Kunming mice and white Leghorn chicken are not endangered animal species.

## Author Contributions

ZJ designed research, performed research, analyzed data, and wrote the paper; XJ cloned and expressed CYP2E1 protein; CL designed research and analyzed data; HX performed animal research and analyzed data; XZ designed research and organized the discussion.

## Conflict of Interest Statement

The authors declare that the research was conducted in the absence of any commercial or financial relationships that could be construed as a potential conflict of interest.The reviewer RY and handling Editor declared their shared affiliation, and the handling Editor states that the process nevertheless met the standards of a fair and objective review.

## References

[B1] Ahmad I. Shukla S. Singh D. Chauhan A. K. Kumar V. Singh B. K. (2014). CYP2E1-mediated oxidative stress regulates HO-1 and GST expression in maneb- and paraquat-treated rat polymorphonuclear leukocytes. *Mol. Cell. Biochem.* 393 209–222. 10.1007/s11010-014-2062-y 24771067

[B2] Chen X. Yin O. Zuo Z. Moses S. (2005). Pharmacokinetics and modeling of quercetin and metabolites. *Pharm. Res.* 22 892–901. 10.1007/s11095-005-4584-1 15948033

[B3] Chen Y. Xianyu Y. Sun J. Niu Y. Wang Y. Jiang X. (2016). One-step detection of pathogens and cancer biomarkers by the naked eye based on aggregation of immunomagnetic beads. *Nanoscale* 8 1100–1107. 10.1039/c5nr07044a 26666198

[B4] Demeilliers C. Maisonneuve C. Grodet A. Mansouri A. Nauyen R. Tinei M. (2002). Impaired adaptive resynthesis and prolonged depletion of hepatic mitochondrial DNA after repeated alcohol binges in mice. *Gastroenterology* 123 1278–1290. 10.1053/gast.2002.35952 12360488

[B5] Dias da Silva W Tambourgi D. V. (2010). IgY: a promising antibody for use in immunodiagnostic and in immunotherapy. *Vet. Immunol. Immunopathol.* 135 173–180. 10.1016/j.vetimm.2009.12.011 20083313 PMC7126787

[B6] Gu L. Gonzalez F. J. Kalow W. Tang B. K. (1992). Biotransformation of caffeine, paraxanthine, theobromine and theophylline by cDNA-expressed human CYP1A2 and CYP2E1. *Pharmacogenetics* 2 73–77. 10.1097/00008571-199204000-00004 1302044

[B7] HaiYing L. NongXue Q. RuiQi Y. WeiHang M. (2009). Correlation analysis between antioxidant capacity and total phenolic content of 86 Chinese edible herbal extracts. *J. Northwest A F University Natl. Sci. Edition* 37 173–180.

[B8] Hartman J. H. Letzig L. G. Roberts D. W. James L. P. Fifer E. K. Miller G. P. (2015). Cooperativity in CYP2E1 metabolism of acetaminophen and styrene mixtures. *Biochem. Pharmacol.* 97 341–349. 10.1016/j.bcp.2015.07.026 26225832 PMC5584065

[B9] He J. Wang Y. Sun S. Zhang X. (2015). Evaluation of Chicken IgY generated against *Canine parvovirus* viral-like particles and development of enzyme-linked immunosorbent assay and immunochromatographic assay for Canine parvovirus detection. *Viral Immunol.* 28 489–494. 10.1089/vim.2015.0030 26469376

[B10] Jiang Z. Chen C. Wang J. Xie W. Wang M. Li X. (2016a). Purple potato (*Solanum tuberosum* L.) anthocyanins attenuate alcohol-induced hepatic injury by enhancing antioxidant defense. *J. Natl. Med.* 70 45–53. 10.1007/s11418-015-0935-3 26481011

[B11] Jiang Z. Chen C. Xie W. Wang M. Wang J. Zhang X. (2016b). Anthocyanins attenuate alcohol-induced hepatic injury by inhibiting pro-inflammation signalling. *Nat. Prod. Res.* 30 469–473. 10.1080/14786419.2015.1020492 25774691

[B12] Jin M. Ande A. Kumar A. Kumar S. (2013). Regulation of cytochrome P450 2e1 expression by ethanol: role of oxidative stress-mediated pkc/jnk/sp1 pathway. *Cell Death Dis.* 4:e554 10.1038/cddis.2013.78 PMC3615729 23519123

[B13] Konstandi M. Cheng J. Gonzalez F. J. (2013). Sex steroid hormones regulate constitutive expression of Cyp2e1 in female mouse liver. *Am. J. Physiol. Endocrinol. Metab.* 304 E1118–E1128. 10.1152/ajpendo.00585.2012 23548611 PMC3651618

[B14] Kovacs-Nolan J. Mine Y. (2004). Avian egg antibodies: basic and potential applications. *Avian Poultry Biol. Rev.* 15 25–46. 10.3184/147020604783637462

[B15] Larsson A. Balow R. M. Lindahl T. L. Forsberg P. O. (1993). Chicken antibodies: taking advantage of evolution–a review. *Poult. Sci.* 72 1807–1812. 10.3382/ps.0721807 8415358

[B16] Li C. He J. X. Ren H. Zhang X. Y. Du E. Q. Li X. P. (2016). Preparation of a chicken scFv to analyze gentamicin residue in animal derived food products. *Anal. Chem.* 88 4092–4098. 10.1021/acs.analchem.6b00426 26980703

[B17] Liang D. L. Zheng S. L. (2014). Effects of icaritin on cytochrome P450 enzymes in rats. *Pharmazie* 69 301–305. 24791596

[B18] Lu K. H. Tseng H. C. Liu C. T. Huang C. J. Chyuan J. H. Sheen L. Y. (2014). Wild bitter gourd protects against alcoholic fatty liver in mice by attenuating oxidative stress and inflammatory responses. *Food Funct.* 5 1027–1037. 10.1039/c3fo60449g 24664243

[B19] Martinez-Gil N. Flores-Bellver M. Atienzar-Aroca S. Lopez-Malo D. Urdaneta A. C. Sancho-Pelluz J. (2015). CYP2E1 in the human retinal pigment epithelium: expression, activity, and induction by ethanol. *Invest. Ophthalmol. Vis. Sci.* 56 6855–6863. 10.1167/iovs.14-16291 26567798

[B20] Nam M. K. Choi H. R. Cho J. S. Cho S. M. Ha K. C. Kim T. H. (2014). Inhibitory effects of Rubi Fructus extracts on hepatic steatosis development in high-fat diet-induced obese mice. *Mol. Med. Rep.* 10 1821–1827. 10.3892/mmr.2014.2398 25050832

[B21] Robertson G. Leclercq I. Farrell G. C. (2001). Nonalcoholic steatosis and steatohepatitis. II. *Cytochrome P*-450 enzymes and oxidative stress. *Am. J. Physiol. Gastrointest. Liver Physiol.* 281 G1135–G1139. 11668021 10.1152/ajpgi.2001.281.5.G1135

[B22] Schade R. Hlinak A. (1995). Egg yolk antibodies, state of the art and future prospects. *Altex* 13 5–9. 11178463

[B23] Shaker E. Mahmoud H. Mnaa S. (2010). Anti-inflammatory and anti-ulcer activity of the extract from *Alhagi maurorum* (camelthorn). *Food Chem. Toxicol.* 48 2785–2790. 10.1016/j.fct.2010.07.007 20633591

[B24] Shayakhmetova G. M. Bondarenko L. B. Voronina A. K. Anisimova S. I. Matvienko A. V. Kovalenko V. M. (2015). Induction of CYP2E1 in testes of isoniazid-treated rats as possible cause of testicular disorders. *Toxicol. Lett.* 234 59–66. 10.1016/j.toxlet.2015.02.008 25683034

[B25] Su T. Mao C. Yin F. Yu Z. Lin Y. Song Y. (2013). Effects of unprocessed versus vinegar-processed *Schisandra chinensis* on the activity and mRNA expression of CYP1A2, CYP2E1 and CYP3A4 enzymes in rats. *J. Ethnopharmacol.* 146 734–743. 10.1016/j.jep.2013.01.028 23376044

[B26] Suzuki K. Nakagawa K. Yamamoto T. Miyazawa T. Kimura F. Kamei M. (2015). Carbon tetrachloride-induced hepatic and renal damages in rat: inhibitory effects of cacao polyphenol. *Biosci. Biotechnol. Biochem.* 79 1669–1675. 10.1080/09168451.2015.1039481 25996516

[B27] Vang O. Jensen H. Autrup H. (1991). Induction of cytochrome P-450IA1, IA2, IIB1, IIB2 and IIE1 by broccoli in rat liver and colon. *Chem. Biol. Interact.* 78 85–96. 10.1016/0009-2797(91)90105-G 2009582

[B28] Wang Y. Ausman L. M. Greenberg A. S. Russell R. M. Wang X. D. (2010). Dietary lycopene and tomato extract supplementations inhibit nonalcoholic steatohepatitis-promoted hepatocarcinogenesis in rats. *Int. J. Cancer* 126 1788–1796. 19551842 10.1002/ijc.24689 PMC2829382

[B29] Wang Y. Wu S. Chen Z. Zhang H. Zhao W. (2015). Inhibitory effects of cytochrome P450 enzymes CYP1A2, CYP2A6, CYP2E1 and CYP3A4 by extracts and alkaloids of *Gelsemium elegans* roots. *J. Ethnopharmacol.* 166 66–73. 10.1016/j.jep.2015.03.002 25764964

[B30] Wargovich M. J. (2006). Diallylsulfide and allylmethylsulfide are uniquely effective among organosulfur compounds in inhibiting CYP2E1 protein in animal models. *J. Nutr.* 136 832S–834S. 16484575 10.1093/jn/136.3.832S

[B31] Xie W. Chen C. Jiang Z. Wang J. Melzig M. F. Zhang X. (2015). *Apocynum venetum* attenuates acetaminophen-induced liver injury in mice. *Am. J. Chin. Med.* 43 457–476. 10.1142/s0192415x15500299 25967663

[B32] Yang J. Qian D. Guo J. Jiang S. Shang E. (2013). Identification of the major metabolites of hyperoside produced by the human intestinal bacteria using the ultra performance liquid chromatography/quadrupole-time-of-flight mass spectrometry. *J. Ethnopharmacol.* 147 174–179. 10.1016/j.jep.2013.02.029 23458919

[B33] Zhou R. Lin J. Wu D. (2014). Sulforaphane induces Nrf2 and protects against CYP2E1-dependent binge alcohol-induced liver steatosis. *Biochim. Biophys. Acta* 1840 209–218. 10.1016/j.bbagen.2013.09.018 24060752 PMC3859691

[B34] Zhou X. Cheung C. M. Yang J. M. Or P. M. Lee W. Y. Yeung J. H. (2015). Danshen (*Salvia miltiorrhiza*) water extract inhibits paracetamol-induced toxicity in primary rat hepatocytes via reducing CYP2E1 activity and oxidative stress. *J. Pharm. Pharmacol.* 67 980–989. 10.1111/jphp.12381 25645193

